# Composite Coating for the Food Industry Based on Fluoroplast and ZnO-NPs: Physical and Chemical Properties, Antibacterial and Antibiofilm Activity, Cytotoxicity

**DOI:** 10.3390/nano12234158

**Published:** 2022-11-24

**Authors:** Dmitriy A. Serov, Dmitriy E. Burmistrov, Alexander V. Simakin, Maxim E. Astashev, Oleg V. Uvarov, Eteri R. Tolordava, Anastasia A. Semenova, Andrey B. Lisitsyn, Sergey V. Gudkov

**Affiliations:** 1 Prokhorov General Physics Institute, Russian Academy of Sciences, 38 Vavilova St., 119991 Moscow, Russia; 2 V. M. Gorbatov Federal Research Center for Food Systems, Russian Academy of Sciences, 26, Talalikhina St., 109316 Moscow, Russia

**Keywords:** zinc oxide nanoparticles, fluoroplast (polytetrafluoroethylene, PTFE), antibacterial activity, antibiofilm activity, surface reconstruction, cytotoxicity, reactive species, meat processing

## Abstract

Bacterial contamination of meat products during its preparation at the enterprise is an important problem for the global food industry. Cutting boards are one of the main sources of infection. In order to solve this problem, the creation of mechanically stable coatings with antibacterial activity is one of the most promising strategies. For such a coating, we developed a composite material based on “liquid” Teflon and zinc oxide nanoparticles (ZnO-NPs). The nanoparticles obtained with laser ablation had a rod-like morphology, an average size of ~60 nm, and a ζ-potential of +30 mV. The polymer composite material was obtained by adding the ZnO-NPs to the polymer matrix at a concentration of 0.001–0.1% using the low-temperature technology developed by the research team. When applying a composite material to a surface with damage, the elimination of defects on a micrometer scale was observed. The effect of the composite material on the generation of reactive oxygen species (H_2_O_2_, •OH), 8-oxoguanine in DNA in vitro, and long-lived reactive protein species (LRPS) was evaluated. The composite coating increased the generation of all of the studied compounds by 50–200%. The effect depended on the concentration of added ZnO-NPs. The antibacterial and antibiofilm effects of the Teflon/ZnO NP coating against *L. monocytogenes*, *S. aureus*, *P. aeruginosa*, and *S. typhimurium*, as well as cytotoxicity against the primary culture of mouse fibroblasts, were studied. The conducted microbiological study showed that the fluoroplast/ZnO-NPs coating has a strong bacteriostatic effect against both Gram-positive and Gram-negative bacteria. In addition, the fluoroplast/ZnO-NPs composite material only showed potential cytotoxicity against primary mammalian cell culture at a concentration of 0.1%. Thus, a composite material has been obtained, the use of which may be promising for the creation of antibacterial coatings in the meat processing industry.

## 1. Introduction

Due to the growth of the world’s population, the food industry is faced with the task of finding new methods to improve the quantity and quality of food products, both of plant and animal origin [1,2]. One of the acute problems is the bacterial contamination of meat and the equipment used at meat processing plants. Even a thorough sanitization, performed under all the rules, cannot completely exclude bacterial contamination of products [3,4]. Contamination of meat processing products with bacteria of epidemiological significance (*Listeria monocytogenes*, *Clostridium perfringens*, *Bacillus cereus*, etc.) is a serious global health problem. For example, in the United States, approximately 17% of the total population suffers from food-borne illness each year. At the same time, more than 128 thousand cases of severe poisoning diseases with the need for hospitalization and 3000 deaths are recorded annually [5]. Among the complications of bacterial infections associated with the intake of meat products, lesions of the gastrointestinal tract (acute poisoning, diarrhea), the central nervous system (meningitis, encephalitis), and the reproductive system (premature birth, stillbirth) are possible [6,7,8,9]. The share of meat products contaminated with bacteria can be a significant part of the total number sold on the market. For example, in China, depending on the type of animal, 22–41% of meat is infected with *B. cereus* [8]. Among pathogens growing on meat products, a significant proportion of antibiotic-resistant strains and strains that can withstand standard disinfection procedures (including treatment with sodium hypochlorite) are found [10].

**Figure 1:**
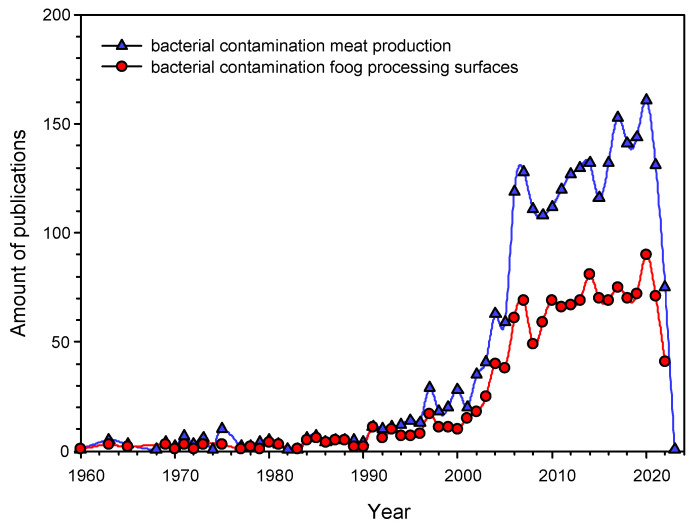
The Number of publications in English addressing the problem of bacterial contamination in the meat industry in general (blue triangles) and bacterial contamination of cutting boards (red circles). The starting point is the first publication on the topic registered at PubMed.

The task of preventing bacterial contamination of meat during butchering is relevant at all stages of its preparation: slaughter, skinning of carcasses and butchering at the enterprise, packaging at points of sale, and final preparation at home [11,12,13]. Referring to the literature, it can be found that the prevention of bacterial contamination of meat at the cutting stage at the enterprise is a key factor in its safety and long-term storage (Figure 1). According to PubMed, over 2300 papers concerning the problem of bacterial contamination of meat products have been published (Figure 1) [https://pubmed.ncbi.nlm.nih.gov accessed 4 October 2022]. Studies devoted to the problem of bacterial contamination of work surfaces (including cutting boards) account for about half of them, >1400 [https://pubmed.ncbi.nlm.nih.gov, accessed on 4 October 2022]. Interest in the study of bacterial contamination of cutting boards increased significantly 20 years ago and has remained high since then (Figure 1). This indicates that there is an active search for ways to solve the problem of bacterial contamination of cutting boards, across the world, although an effective solution has not yet been found.

There are several approaches being developed to protect cutting boards from bacterial contamination. The first approach is to physically remove the contaminated layer of the cutting board by sanding or cutting off the top layer [14]. This method is widely used at present, particularly for cutting boards made of polymeric materials, but its disadvantage is the need for frequent cleaning, which causes accelerated wear of the cutting boards. The second approach is the modification of disinfection protocols and the use of combined sterilization methods to remove already attached bacteria [15,16]. Unfortunately, the use of combinations of methods can significantly increase the price of the finished product, and in the case of combinations of aggressive chemical compounds, more thorough removal of disinfecting agents after processing will be required. In addition, in the case of the formation of biofilms in microdamages, bacteria can acquire a tolerance of chemical compounds used for disinfection [17,18]. The third approach is to cover working surfaces with protective films based on oils (Food-Safe Oil Coating) or polymeric materials to prevent bacterial adhesion [19,20,21]. This method can significantly reduce the number of attached bacteria due to the removal of irregularities and microdamages [22,23]. Additional advantages of this approach are the increase in the service life of cutting boards and the absence of the need for complex and expensive methods of disinfection. However, the process of applying Food-Safe Oil Coating is a multi-stage process [19]. For industrial applications, it is highly desirable to develop methods for coating cutting boards with a minimum number of steps (preferably up to one).

Fluoroplast (polytetrafluoroethylene, PTFE) or a polymeric resin composed of tetrafluoroethylene monomers forms an opaque and smooth milky white material. Fluoroplast is widely used for many purposes: the production of bearings and gaskets in mechanical engineering, increasing the efficiency of chemical production, the manufacture of filters for water and air purification and the coating of chemical vessels and kitchen utensils [21,24,25,26,27,28,29,30]. It has good chemical, thermal and electrical stability and a low friction coefficient [31]. An additional advantage of fluoroplast is its very low toxicity to mammals. In the case of oral intake of fluoroplast microparticles into the body of mice, there are no significant toxic effects in vivo [32]. Given these advantages, fluoroplast coatings are already being developed for use in the food industry to reduce the adhesion of working surfaces [21,33]. We believe that to achieve the best protection of cutting boards from bacterial contamination, it is necessary not only to reduce the adhesion of bacteria to the material of the boards but also to acquire active antimicrobial properties for the materials. As mentioned earlier, many strains of bacteria inhabiting cutting boards are antibiotic-resistant [34,35]; therefore, alternative methods of combating bacterial contamination are currently being sought. One of the most promising methods is the use of nanoparticles (NPs) of metals and their oxides. For metal oxide NPs, the ability to inhibit the growth of epidemiologically significant bacterial strains, including antibiotic-resistant ones, has been described [36,37,38]. Several mechanisms of the antibacterial action of metal oxide NPs have been described: impaired functioning of proteins and enzymes [39], disruption of bacterial DNA replication [40,41], the genotoxic effect [42,43], inhibition of ATP synthesis [44], damage of cell walls and bacterial membranes [45,46,47], increased oxidative stress [40,44,48], photocatalytic activity [49,50,51]. Among metal oxide NPs, ZnO-NPs has a pronounced bactericidal and antifungal effects [36,52,53,54]. In some cases, the minimum inhibitory concentration (MIC) of ZnO-NPs is ~1 μg/mL [55,56]. Combining a polymer matrix and metal oxide NPs can be a new step in the creation of materials with the desired physical properties and protection against bacterial contamination. We have previously obtained materials based on polymeric matrices of borosiloxane or PLGA and nanoparticles of silver, aluminum, iron, and zinc oxides [57,58,59,60]. The previously obtained materials possessed good antimicrobial activity. We assume that the combination of fluoroplast and ZnO-NPs will make it possible to obtain a material that, on the one hand, is strong enough to protect working surfaces from micro damage and, on the other hand, has its bacteriostatic activity. Undoubtedly, it is impossible to completely avoid classical methods of sterilization. We hope that our results will make it possible to minimize the number of chemical agents used

The study aims to develop a composite material based on fluoroplast and ZnO-NPs, to study its physicochemical properties, antimicrobial activity, and biocompatibility with eukaryotic cells.

## 2. Materials and Methods

### 2.1. ZnO-NPs Synthesis and Characterization

The method of laser ablation in a liquid was used for the synthesis of the ZnO-NPs. A pulsed ytterbium-doped fiber laser was used. Laser radiation parameters: λ = 1064 nm, τ = 4–200 ns; ν = 20 kHz; average power up to 20 W; E = 1 mJ. The liquid layer (0.05 M NaNO_3_ aqueous solution) over the target (metal zinc plate) was about 1 mm. Irradiation time varied in the range of 5–20 min. Using the Zetasizer Ultra Red Label (Malvern Panalytical Ltd., Malvern, UK), the hydrodynamic diameter (DLS) and the zeta potential (ELS) of the obtained NPs were determined. The NP diameter was confirmed using a CPS 24000 (CPS Instruments, Prairieville, LA, USA) [61]. The morphological features (shape, topology) and the elemental composition of the NPs, were studied using a Libra 200 FE HR transmission electron microscope in combination with a JED-2300 energy dispersive X-ray spectrometer (Carl Zeiss, Jena, Germany). The composition of the obtained colloidal solutions of NPs was confirmed using Cintra 4040 (GBC Scientific Equipment, Braeside, Australia). Samples preparation for TEM was performed according to the following protocol. A gold mesh Ø ~4 mm was placed in a titanium holder. Samples of ZnO-NPs colloids were applied to the grid as 0.25 µL drops. The samples were dried at room temperature for 10 min with subsequent evacuated.

### 2.2. Preparation and Characterization of the Composite Material

After the synthesis of the nanoparticles, water was replaced with acetone by centrifugation. The colloidal solution of the nanoparticles was centrifuged in a Sigma 3-16KL centrifuge (Sigma Laborzentrifugen GmbH, Osterode am Harz, Germany) with a 12158 rotor for 40 min at 7000× *g*; then, the supernatant was carefully replaced with acetone. These manipulations were carried out 3 times. Next, the resulting colloidal solution was mixed with a fluoroplastic varnish to a final concentration of nanoparticles of 0.1, 0.001, and 0.0001%. The varnish is a fluoroplastic dissolved in a mixture of acetone, butyl acetate, cyclohexanone, and toluene in a ratio of 25:40:10:25 mass parts. Drops of a solution of nanoparticles in lacquer with a volume of 500 μL were applied to round degreased 25 mm glasses. Before the start of the experiments, the coatings were dried for 48 h in a fume hood. For cytotoxic and microbiological studies, samples coated with a composite coating were pre-disinfected by soaking in 70% ethanol for 2–3 h. For microbiological studies, the varnish was applied to cubes (4 × 4 × 6 mm). The coating microstructure was studied using an NPX200 atomic force microscope (Seiko Instruments, Tokyo, Japan) [62]. The distribution of nanoparticles in the fluoroplast polymer was assessed using a MIM-321 modulation-interference microscope (Amphora Lab, Moscow, Russia).

### 2.3. Quantification of ROS Concentration

The concentration of hydrogen peroxide formed in aqueous solutions was estimated from the intensity of the chemiluminescence of the luminol-p-iodophenol-horseradish peroxidase system. The chemiluminescence was measured on a highly sensitive Biotox-7A-USE chemiluminometer (Engineering Center—Ecology, Moscow, Russia). Samples of the studied composite material were placed in polypropylene vials (Beckman, Brea, CA, USA) with the addition of 1 mL of a “counting solution”, prepared immediately before the measurement, containing 1 mM Tris-HCl buffer pH 8.5, 50 μM p-iodophenol, 50 μM luminol, 10 nM horseradish peroxidase. The sensitivity of this method is <1 nM [63]. To quantify the content of hydroxyl radicals in aqueous solutions, the reaction with coumarin-3-carboxylic acid (CCA) was used. The hydroxylation reaction produces the fluorescent product 7-hydroxycoumarin-3-carboxylic acid (7-OH-CCA). Fluorescence of 7-OH-CCA) was recorded using a JASCO 8300 spectrofluorimeter (JASCO, Tokyo, Japan) at λ_ex_ = 400 nm, λ_em_ = 450 nm.

### 2.4. Measurement of the Concentration of the Formed Active Long-Lived Forms of Proteins

The number of long-lived reactive forms of proteins was estimated using the chemiluminescence of the protein solutions after heating to 40 °C for 2 h. All samples were stored in the dark at room temperature for 30 min after exposure. The measurements were carried out in 20 mL polypropylene vials (Beckman, Brea, CA, USA) in the dark at room temperature on a highly sensitive Biotox-7A chemiluminometer (Engineering Center—Ecology, Moscow, Russia). Protein solutions not subjected to heating were used as controls [64].

### 2.5. Quantitative Determination of 8-Oxoguanine in DNA In Vitro by ELISA

To quantify 8-oxoguanine in DNA, a non-competitive enzyme-linked immunosorbent assay (ELISA) was developed using monoclonal antibodies specific for 8-oxoguanine (anti-8-OG antibodies). DNA samples (350 μg/mL) were denatured by boiling in a water bath for 5 min and cooling on ice for 3–4 min. Aliquots (42 μL) were applied to the bottom of the wells of the ELISA plates. DNA was immobilized using a simple adsorption procedure with incubation for 3 h at 80°C until the solution was completely dry. Non-specific adsorption sites were blocked with 300 μL of a solution containing 1% skimmed milk powder in 0.15 M Tris-HCl buffer, pH 8.7, and 0.15 M NaCl. Then, the plates were incubated at room temperature overnight (14–18 h). The formation of an antigen-antibody complex with anti-8-OG antibodies (at a dilution of 1: 2000) was carried out in a blocking solution (100 μL/well) by incubation for 3 h at 37 °C. Washed twice (300 μL/well) with 50 mM Tris-HCl buffer (pH 8.7) and 0.15 M NaCl with 0.1% Triton X-100 after 20 min incubation. Next, a complex was formed with a conjugate (anti-mouse immunoglobulin labeled with horseradish peroxidase (1:1000)) by incubation for 1.5 h at 37 °C in a blocking solution (80 μL/well). The wells were then washed 3 times, as described above. A chromogenic substrate containing 18.2 mM ABTS and hydrogen peroxide (2.6 mM) in 75 mM citrate buffer, pH 4.2 (100 μL/well) was added to each well. Reactions were stopped by adding an equal volume of 1.5 mM NaN_3_ in 0.1 M citrate buffer (pH 4.3) when color was achieved. The optical density of the samples was measured on a tablet spectrophotometer (Titertek Multiscan, Helsinki, Finland) at λ = 405 nm. The method was described in more detail earlier [65].

### 2.6. Evaluation of Antibacterial Activity and Antibiofilm Activity of Samples

The antibacterial properties of fluoroplast/ZnO-NPs coatings containing various concentrations of ZnO-NPs were tested against 2 g-positive (*Listeria monocytogenes*, *Staphylococcus aureus*) and 2 g-negative (*Pseudomonas aeruginosa*, *Salmonella typhimurium*) bacterial species. Bacterial cultures were obtained from the working collection of the Laboratory of Microbiology, Research Institute of Food Systems named after Gorbatov. A Luria-Bertani (LB) medium (BD Difco, Franklin Lakes, NJ, USA) and a tryptone soy broth (TSB) (Panreac AppliChem, Barcelona, Spain) were used as culture media. As test surfaces for culturing bacterial cells, we used Teflon cubes with sides 4 × 4 × 6 mm, coated with a composite material with various concentrations of ZnO-NPs (0.001–0.1%) in the composition, as well as without coating (Control−) and with fluoroplast coating, not containing ZnO-NPs (Control+). For research, the daily broth culture of the studied microorganism was diluted 100 times in sterile broth (LB) and poured into sterile test tubes (V = 2 mL). Next, pre-sterilized Teflon cubes (one sample each) were added to each test tube and incubated for 6 and 18 h in a thermostat, at a temperature of 37 °C. After incubation, the cubes were washed once in distilled water (to remove planktonic cells), transferred into test tubes with sterile saline (0.9% NaCl solution), and vigorously shaken 3 times for 15 min. Further, the obtained washings were titrated (tenfold dilutions were carried out), transferred to Petri dishes with a dense nutrient medium, and evenly distributed over the surface with a sterile spatula. The results were recorded by counting the number of colony-forming units (CFU) 24 h after incubation at 37 °C. To study the antibiofilm activity of fluoroplast/ZnO-NPs coatings, a broth culture of *Pseudomonas aeruginosa* V = 30 µL was applied to the surface of Teflon cubes and left to dry at room temperature for 30 min. Further, to visualize living and dead cells, the samples were stained with a set of fluorescent dyes Filmtracer Live/Dead Biofilm Viability Kit (Invitrogen, Waltham, MA, USA) and viewed under a microscope with appropriate filters. This kit contains SYTO^®^9 fluorescent dyes and propidium iodide (PI). Both of them stain the DNA of microorganisms, however, SYTO^®^9 can quickly penetrate the membrane of living bacteria, while propidium iodide (PI) has a harder time penetrating the wall of living bacteria. After 20 min of staining, living cells are visualized in green and dead cells in red. Microscopy was performed using the Eclipse Ni imaging system (Nikon, Tokyo, Japan).

### 2.7. Isolation and Cultivation of Fibroblasts from Mouse Lungs

All manipulations with animal tissues and cells were carried out in clean rooms using a Laminar-S class II biological safety cabinet (Lamsystems, Miass, Russia). Primary cell cultures of isolated fibroblasts from mouse lungs were obtained according to a standard protocol with minor modifications. Euthanasia of a 2–3-month-old mouse was performed by displacing the cervical vertebrae. With the help of surgical scissors, the lungs were removed from the chest. The lungs were placed in a sterile Petri dish ⌀60 (TPP, Switzerland) containing a small volume of PBS solution. The organs were chopped with sterile scissors into pieces with a volume of ~1 mm^3^. Pieces of lung tissue were incubated for 1 h in 25 mL of DMEM medium containing 0.2% (*w*/*v*) type II collagenase at 37 °C on an MR-1 rocking shaker (Biosan, Riga, Latvia). Collagenase was inhibited with 20% FBS. Tissue pieces incubated in collagenase solution were resuspended by pipetting and then passed through an EASTstrainer™ sieve with a mesh size of 70 µm (Greiner bio-one, Kremsmunster, Austria). Cells were washed by double centrifugation at 350× *g* for 5 min in DMEM. The isolated cells were further cultured in TC T-25 culture mats (TPP, Trasadingen, Switzerland) in DMEM/F12 medium supplemented with 10% FBS, 2 mM L-glutamine, 100 U/mL penicillin, 100 μg/mL streptomycin obtained from the PanEco campaign (PanEco, Moscow, Russia). Upon reaching 80–90% confluence, the cells were adhered with 0.05% Trypsin-EDTA solution (PanEco, Moscow, Russia) for 5 min at 37 °C. Trypsin was inactivated with 10% FBS. Before starting cytotoxic studies, the cells were passaged at least 3 times.

### 2.8. Cytotoxicity Study on Mouse Lung Fibroblasts

Round glasses for microscopy ⌀25 coated with fluoroplast/ZnO-NPs and without (control group) composite coating (V = 500 µL) were sterilized in 70% ethanol for 2–3 h and then placed in wells of 6- well plate (TPP, Trasadingen, Switzerland). A cell suspension (50 μL) was applied to each coating sample, then incubated for 45 min for cell adhesion, after which it was brought to a final volume of 1 mL with a warm culture medium. The total time from the moment the cells were planted on the surface to microscopic measurements was at least 72 h. Cultivation was carried out in a CO_2_ incubator (Biosan, Riga, Latvia) at 37 °C and 5% CO_2_. DMEM/F12 with additives was used as a medium for cell cultivation, the preparation of which was described in detail by the research team earlier. The initial number of cells in the suspension placed on the surface of the material was ~50,000 cells per sample. Hoechst 33342 dyes and propidium iodide (PI) were used to evaluate the cytotoxicity. Immediately after incubation, the coating sample with cultured cells was placed in a cover slip chamber (RC-40LP, Warner Instruments, Holliston, MA, USA), thoroughly washed with PBS, stained with Hoechst 33342 at a concentration of 5 µg/mL, and incubated for 30 min at 37 °C. Then, the sample was washed with PBS and stained with 2 μM PI (ThermoFisher, Waltham, MA, USA) for 1 min. The samples were analyzed using a DMI4000 B fluorescence microscope (Leica Microsystems, Wetzlar, Germany) equipped with an SDU-285 digital camera (SpetsTeleTekhnika, Moscow, Russia). Fluorescence spectra were recorded at excitation/emission wavelengths: 350/470 for Hoechst 33342 (D filter cube, Leica, Germany), 540/590 for PI (TRITC Leica filter cube, Wetzlar, Germany). Light emitting diodes (LED) M375D2m, M490D3 (Thorlabs, Newton, NJ, USA) and white LED (Cree Inc., Durham, NC, USA) were used as light sources for the excitation of Hoechst 33342 and PI fluorescence. All images were taken at the following LED currents: 100 mA for M375D2m LED (Hoechst 33342) and 250 mA for white LED (PI). The exposure time in all experiments was the same: 500 ms for Hoechst 33342 and 700 ms for PI. The detector gain was ×423 and was the same for all fluorophores and experimental conditions.

Data acquisition and microscope setup control were performed using Win-FluorXE software (J. Dempster, Strathclyde Electrophysiology Software, University of Strathclyde, UK). The data were collected as 12-bit grayscale images. Subsequent analysis was performed using ImageJ2 (Fiji) software (NIH, Bethesda, MD, USA). For each variant of the experiment, at least five samples were analyzed. At least 200 cells were analyzed in each sample. ROI was determined using ImageJ’s “Threshold” and “Particle Analysis” automated procedures. For an image of 1392 × 1024 pixels, obtained with a value of ×20, the following parameters were used: “size” = 100–750 and “roundness” = 0.10–1.00. The nuclei had different fluorescence intensities from Hoechst 33342 and PI. To ensure that all nuclei are included in the analysis, we performed a series of thresholding and particle analysis procedures on each image. The images were converted to 8-bit before determining the ROI. The threshold levels ranged between 5 and 255 a.u. with a step of 5 c.u. The ROIs were saved as binary masks, then all of the ROIs were combined with the removal of duplicates and all procedures were integrated into automated macros.

## 3. Results

**Figure 2:**
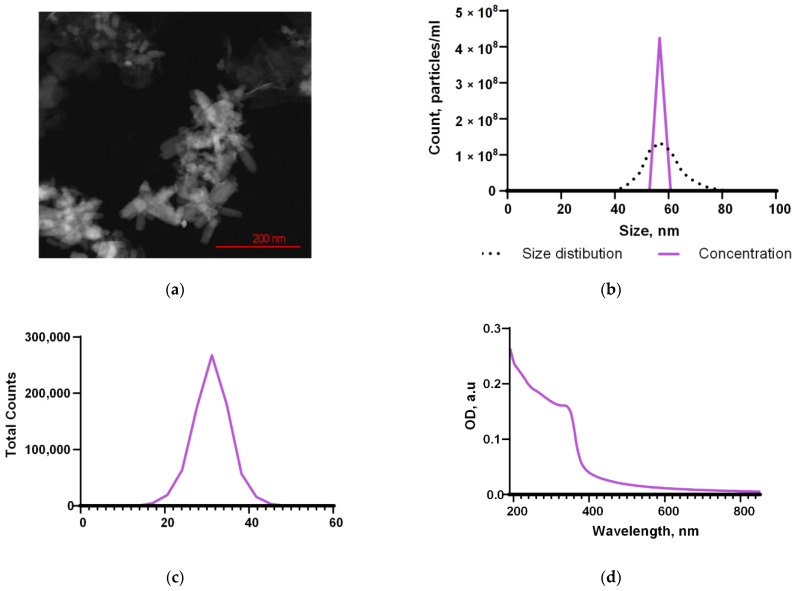
Physical properties of the synthesized NPs. The TEM image of a group of nanoparticles (**a**); the concentration and the size distribution of nanoparticles (**b**); the ζ-potential distribution (**c**); the optical absorption spectrum (**d**).

The ZnO nanoparticles synthesized by laser ablation had a rod-like morphology, determined by TEM (Figure 2a). The average NP size, according to the TEM data, was in the range of 30–80 nm (Figure 2a). Using DLS, it was found that the average concentration of NPs in the colloid was ~4.2 × 10^8^ mL^−1^ (Figure 2b). The range of hydrodynamic diameters of the obtained NPs, determined by the DLS method, was 40–80 nm; the average NP size was ~57 nm (Figure 2b). Using ELS, the distribution of the ζ-potential of the nanoparticles was estimated. The NP’s ζ-potential values ranged between 20 and 40 mV with a maximum of 30 mV (Figure 2c). The characteristic absorption spectrum of the NP colloid suggests that NPs are predominantly composed of zinc oxide (Figure 2d).

**Figure 3:**
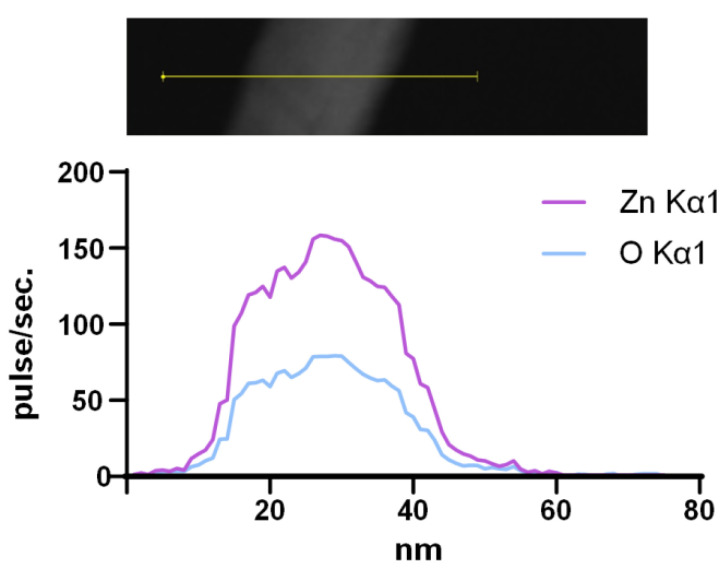
Elemental composition of synthesized ZnO-NPs. Analyzed region of NPs (image on the top) and profile of Zn Kα1 and O Kα1 (spectrum on the bottom).

It is important to establish the exact chemical composition and purity of synthesized ZnO-NPs. The characteristic optical spectrum is indirect evidence of the chemical composition of nanoparticles. The obtained EDS spectra made it possible to determine directly that the composition of the obtained NPs contains only Zn and O atoms. The spectra of other elements were not detected; therefore, ZnO-NPs have a high degree of purity (Figure 3).

**Figure 4:**
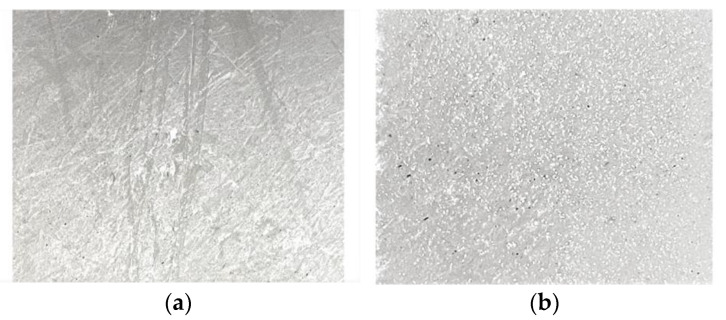
Photograph of the damaged Teflon sample before (**a**) and after (**b**) application of the fluoroplast/ZnO-NPs composite material.

A composite material based on fluoroplast/ZnO-NPs was deposited on the surface of a damaged Teflon bar (damage depth <1 mm). The fluoroplast/ZnO-NPs composite material was found to uniformly cover the damaged surface of the Teflon bar (Figure 4a) and also fill in any visible defects present (Figure 4b).

**Figure 5:**
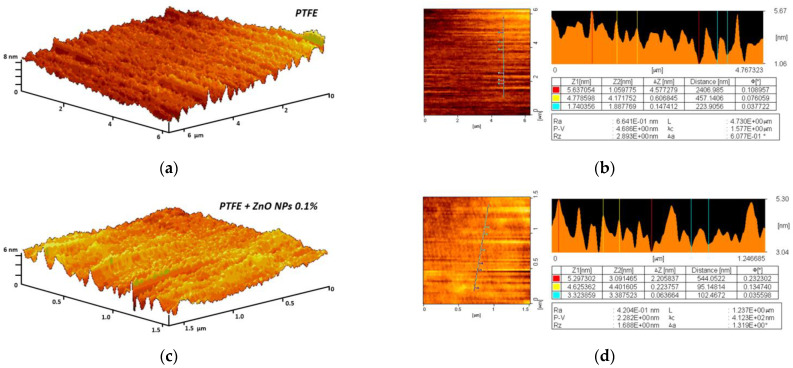
Evaluation of composite material surface inhomogeneity using the AFM method: 3D reconstruction of the fluoroplast coating surface without ZnO-NPs (**a**) and with the addition of 0.1% ZnO-NPs (**c**); Examples of the results of a quantitative assessment of the surface heterogeneity of a fluoroplast coating without ZnO-NPs (**b**) and with the addition of 0.1% ZnO-NPs (**d**).

Using atomic force microscopy, we found that the polymerized composite coating without NPs and after the addition of ZnO-NPs, even at a concentration of 0.1%, has no recorded defects (breaks, creases, grooves) at the micro level (Figure 5a,c). In the analyzed areas, the surface inhomogeneity did not exceed a few nm (Figure 5b,d).

**Figure 6:**
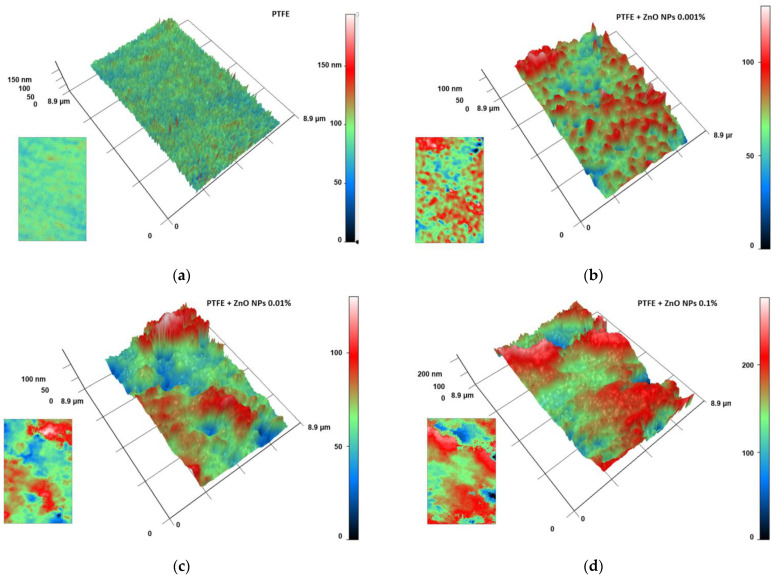
Estimation of the distribution of ZnO-NPs inside the composite material: MIM results for fluoroplast without NPs (**a**); with the addition of ZnO-NPs 0.001% (**b**); ZnO-NPs 0.01% (**c**) or ZnO-NPs 0.1% (**d**). The data are presented as 3D reconstructions, where Ox and Oy are real distances in µm and Oz is the phase difference expressed in nm. The original phase difference maps are shown as insets in the lower left corner of the corresponding panel.

The MIM method was used to evaluate the distribution of NPs inside the polymer matrix. In fluoroplast, without the addition of NPs, no regions with a significant phase difference were found. The phase difference within the vast majority of the analyzed polymer region did not exceed tens of nm, on average (Figure 6a). When the ZnO-NPs were added at a concentration of 0.001%, regions with a significant phase difference were found. These areas may indicate a locally increased density of NPs or the presence of aggregates of ZnO-NPs inside the polymer matrix. The approximate sizes of the zones with high phase change were 0.1–0.5 µm (Figure 6b). With an increase in the concentration of introduced NPs to 0.01%, an increase in the sizes of such zones up to 0.2–2.0 µm was observed. The phase difference was 100–150 nm (Figure 6c). The addition of ZnO-NPs at a concentration of 0.1% caused the bands to increase to 1.0–4. µm. In addition, an increase in the phase difference to 200–240 nm was observed (Figure 6d).

**Figure 7:**
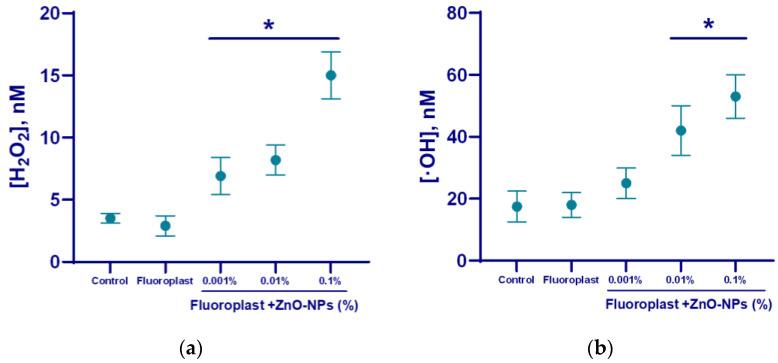
Effect of fluoroplast/ZnO-NPs composite coating on ROS generation in aqueous solutions: hydrogen peroxide (**a**), hydroxyl radicals (**b**). *—statistically significant differences compared to the control group (*p* < 0.05). Data are presented as mean ± standard error of the mean.

The ability of the composite material to influence the generation of ROS was studied (Figure 7). Fluoroplast without the addition of NPs did not affect the production of either hydrogen peroxide (Figure 7a) or hydroxyl radical (Figure 7b). The concentrations of hydrogen peroxide and hydroxyl radical in the control were 5 and 20 nM, respectively (Figure 7). The addition of the ZnO-NPs at concentrations of 0.001, 0.01, and 0.1% in fluoroplast increased the generation of hydrogen peroxide by 50, 60, and 200%, respectively, compared to fluoroplast without NPs (Figure 7a). The addition of the ZnO-NPs at concentrations of 0.01 and 0.1% increased the generation of hydroxyl radicals by 100 and 150% proper (Figure 7b). It should be noted that the introduction of 0.001% ZnO-NPs into the polymer did not affect the production of hydroxyl radicals compared to fluoroplast without NPs.

**Figure 8:**
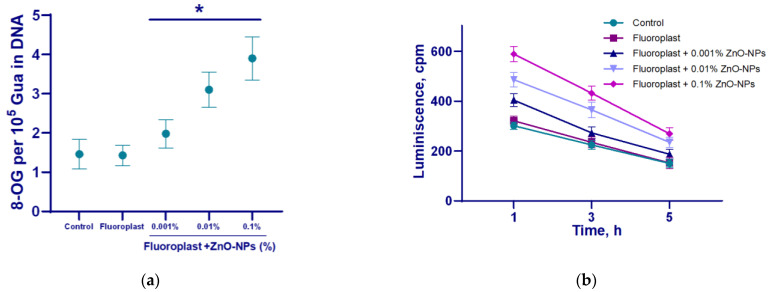
The effect of the fluoroplast/ZnO-NPs composite coating on the formation of 8-OG in DNA (**a**) and the dynamics of the formation of active long-lived forms of proteins (**b**). *—statistically significant differences compared to the control group (*p* < 0.05). Data are presented as mean ± standard error of the mean.

To evaluate the potential effect of the resulting composite material on biopolymers, we studied the effect of fluoroplast/ZnO-NPs on the production of a key biomarker of DNA damage, 8-oxoguanine, and the generation of long-lived active forms of proteins. In the uncoated control, 8-oxoguanine generation was ~1.5/10^5^ DNA guanines (Figure 8a). The intensity of the LRPS chemiluminescence in the control was no more than 300 cpm. The half-life of LRPS is ≥5 h (Figure 8b). We found that NP-free fluoroplast did not affect the formation of 8-oxoguanine (Figure 8a) and LRPS (Figure 8b). The addition of ZnO-NPs increased the production of 8-oxoguanine in DNA in a concentration-dependent manner. The addition of the highest concentration of NPs (0.1%) increased the production of 8-oxo-guanine and LRPS by more than two times. The ZnO-NPs did not affect the half-life of LRPS (Figure 8b).

**Figure 9:**
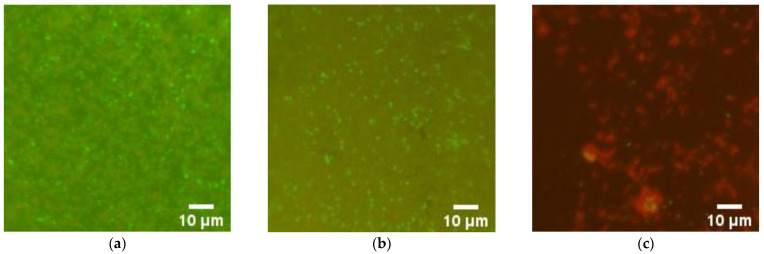
Representative microphotographs of bacterial colonies stained with SYTO^®^9 (viable—green cells) and PI (dead—red cells) detected on the surface of Teflon (Control−) (**a**), coating free of ZnO-NPs (Control+) (**b**), fluoroplast/ZnO-NPs composite coating with 0.1% ZnO-NPs (**c**).

To evaluate the bacteriostatic (Table 1) and bactericidal (Figure 9) effect of the composite material, a microbiological study was carried out. Polymer material fluoroplast without ZnO-NPs did not affect the growth of all studied bacteria (Table 1) after 6 and 18 h of incubation. The addition of 0.001% ZnO-NPs significantly inhibited the growth of all studied microorganisms. After 6 h of incubation, no more than 12 CFU/mL was found in all samples. After 16 h, the number of CFU/mL of gram-negative *P. aeruginosa* and *S. typhimurium*, and gram-positive *L. monocytogenes* and *S. aureus* was reduced by three orders of magnitude compared with the control. On the fluoroplast/0.01% ZnO-NPs composite material, after 6 h, there were no studied microorganisms observed. After 18 h of incubation on a fluoroplast/0.01% ZnO-NPs composite, the number of Gram-negative bacteria *P. aeruginosa* and *S. typhimurium* decreased by two orders of magnitude, and of Gram-positive bacteria, *L. monocytogenes* and *S. aureus*, by three orders of magnitude, compared with the control. On the fluoroplast/0.1% ZnO-NPs composite material, after 6 h, no CFU of all four studied bacterial strains was detected. After 16 h, the number of CFU of all microorganisms did not exceed ≤5. The microbiological study performed showed that the fluoroplast/ZnO-NPs coating has strong bacteriostatic effects against both Gram-positive (*L. monocytogenes*, *S. aureus*) and Gram-negative bacteria (*P. aeruginosa*, *S. typhimurium*) (Table 1). At the same time, the bacteriostatic effect slightly depended on the Gram affiliation of the bacteria.

Using fluorescence microscopy, the effect of the composite material on the destruction of biofilms was studied. The grown biofilms were in contact with the composite material for several hours. It was found that the uncoated surface of the cutting board and areas coated with fluoroplast and the composite material with ZnO NPs have background illumination, but it did not interfere with the visualization of microorganisms (Figure 9). It was found that fluoroplast without NPs did not affect the viability of the bacterial cells that make up the biofilm. In this case, the contact of the biofilm with the composite material based on fluoroplast/ZnO-NPs 0.1% leads to almost complete non-viability of the biofilm cells.

Notably, most of the dead cells found on the surface of the fluoroplast/ZnO-NPs composite coating had abnormal morphology (Figure 9c). Thus, the fluoroplast/ZnO-NPs composite coating functionalized with 0.1% ZnO-NPs had clear antibacterial properties and also prevented biofilm growth.

**Figure 10:**
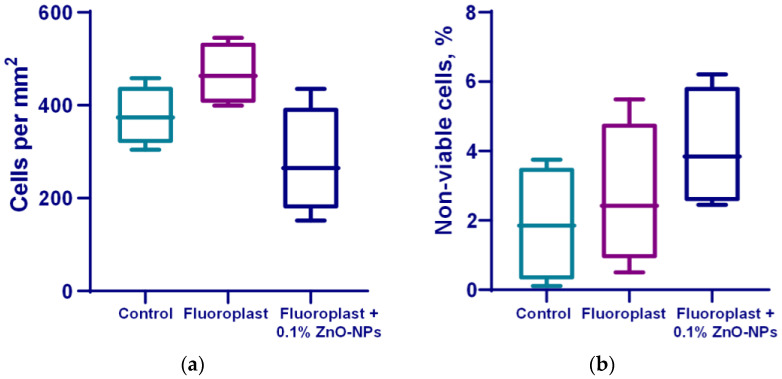

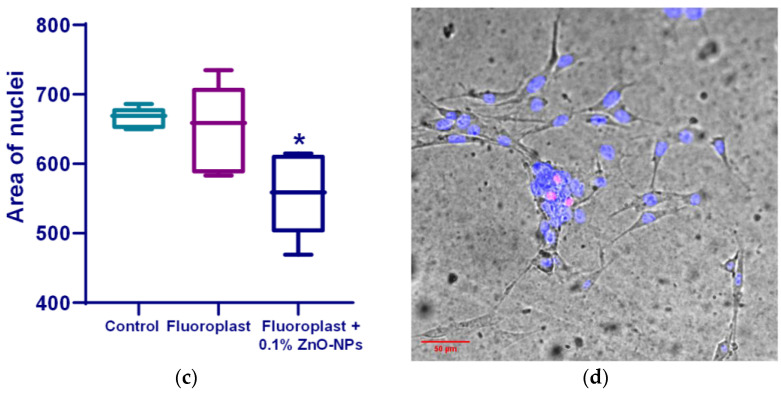
Effect of fluoroplast/ZnO-NPs composite coating on in vitro mouse fibroblast growth at 4 DIV. Density of cell cultures (**a**); percentage of non-viable cells (**b**); average area of nuclei (**c**); representative microphotograph of cells growing on the surface of a composite coating based on fluoroplast containing 0.1% ZnO-NPs (Hoechst, PI, transmitted light merge) (**d**). *—statistically significant differences compared to the “Control” group (*p* < 0.05). Data are presented as mean ± standard error of the mean.

Cytotoxic studies on the mouse lung fibroblast cells showed that on the surface, fluoroplast coatings without ZnO-NPs did not affect cell growth and development; the density of the cell cultures was higher than that of the cultures growing on the glass. At the same time, the fluoroplast/ZnO-NPs composite coating containing 0.1% NPs exhibited potential cytotoxicity. There was a trend towards a decrease in the density of the cell cultures compared to the cultures growing on the surface of fluoroplast without NPs (Figure 10a). The percentage of non-viable cells by day four of in vitro culture also tended to increase (Figure 10b). At the same time, the average area of the cell nuclei decreased by ~14% compared to the control group (Figure 10c). It is important to note that there were no obvious morphological changes in the cells growing on the coating with ZnO-NPs (Figure 10d).

## 4. Discussion

Using laser ablation, we obtained nanoparticles of zinc oxide. This method makes it possible to synthesize stable colloidal solutions of metal nanoparticles and metal oxides and does not require the use of blocking and stabilizing agents or reducing agents; this ensures the production of nanoparticles with the required morphological parameters [66,67]. The resulting nanoparticles had an average size of approximately 60 nm, confirmed by TEM and DLS (Figure 2a,b). The maximum of the ζ-potential was 30 mV, which was confirmed by ELS (Figure 2c). The value of the ζ-potential makes it possible to predict the stability of the colloidal system. NPs with values >+25 mV or <−25 mV usually have a high degree of stability. Lower values of the ζ potential lead to aggregation or flocculation due to van der Waals interactions between particles [68]. We also evaluated the morphology of the synthesized ZnO nanoparticles (Figure 2a). Using TEM, it was found that the nanoparticles have the morphology of nano-rods. In general, the morphology of nanomaterials can influence some properties, including antimicrobial activity, by the increase in the surface area. Several studies have shown that ZnO nanoparticles with spike-like morphology exhibited antibacterial and antifungal activity against *B. subtilis*, *E. coli*, and *C. albicans* [52,69].

The obtained NPs consist mainly of ZnO (Figure 2d and Figure 3). The main mechanism for the implementation of the antibacterial action of nano-sized zinc oxide is the release of Zn^2+^ ions from the medium [70]. In addition, as a result of the photocatalytic reactions on the surface of the ZnO nanoparticles, the formation of such ROS as hydroxyl radical, superoxide anion radical and hydrogen peroxide is possible. The hydroxyl radical has an extremely short half-life (10^–9^ s) and a small diffusion radius and has high reactivity, which makes this compound very dangerous for the body. H_2_O_2_, on the contrary, is a relatively stable compound, has a large diffusion radius, and can pass through the cell membrane [71]. It was found that the obtained fluoroplast/ZnO contributed to the generation of hydroxyl radicals and hydrogen peroxide (Figure 5 and Figure 6). An increase in the rate of generation of hydrogen peroxide by ~3 times and hydroxyl radicals by 3 times were noted for the coating containing 0.3% NPs in the composition. It is known that high above-threshold concentrations of ROS cause oxidative stress in cells, which is accompanied by damage to membrane structures, oxidation of proteins, and DNA. Bacterial cells are vulnerable to high doses of ROS, despite the antioxidant system formed during evolution [72]. In the course of the oxidative action of ROS, compounds are formed that lead to a violation of the functional integrity of the biopolymer, such as lipid peroxidation products that affect the phase state of the lipid bilayer and modify the conductivity of membranes for ions and small molecules; fragmentation, breaks, and modifications of DNA, in particular, the formation of 8-oxoguanine in DNA, leading to mismatches of nucleotides for adenine; the formation of active forms of proteins that promote the formation of secondary free radicals. In this regard, we considered the ability of fluoroplast/ZnO-NPs coatings to cause oxidative damage to proteins (Figure 8b), as well as to DNA with the formation of 8-oxoguanine (Figure 8a). It was found that the tendency to form 8-oxoguanine in DNA in vitro was found in the composite with the lowest content of ZnO-NPs (0.001%) and increased in proportion to the increase in the concentration of NPs in the composition, reaching 3.9 per 10^5^ guanines in DNA at a ZnO-NPs concentration of 0.1%. (Figure 8a). An increase in the rate of formation of long-lived reactive forms of proteins was also found, with a half-life of approximately 5 h (Figure 8b).

Microbiological studies on four types of microorganisms: *Pseudomonas aeruginosa*, *Salmonella typhimurium*, *Listeria monocytogenes* and *Staphylococcus aureus* showed that the applied fluoroplast/ZnO-NP coating over bulk Teflon provided a bacteriostatic effect at NP concentrations of 0.001 and 0.01% for 18 h of cultivation (Table 1). At the same time, the Gram-positive bacteria *Listeria monocytogenes* and *Staphylococcus aureus* showed a higher sensitivity to the composite material compared to the other species under consideration. It is noteworthy that some studies have demonstrated a different antibacterial effect of nanomaterials based on metal oxides concerning Gram-positive bacteria compared to Gram-negative bacteria. Both types of bacteria have a negatively charged cell wall due to teichoic acids in their composition. It is assumed that the negative potential of the bacterial cell surface is an important factor in the interaction between NPs, as well as ions released from NPs, and the bacterial cell [73]. It is important to note that the cultivation of the considered bacterial cells on the surface of the fluoroplast containing ZnO NPs at a concentration of 0.1% revealed a clear bactericidal effect, accompanied by the almost complete absence of viable bacterial cells of all considered bacterial species after 18 h of cultivation; at the same time, most of the dead cells, with the help of microscopy, revealed abnormal morphology (Figure 9). A comparison of the antibacterial activity of the fluoroplast/ZnO NP composite developed by our research team with the composites described in the literature and our previous data is shown in Table 2. It should be noted that, in most studies, the antibacterial activity is assessed by the severity of the bacteriostatic effect [74,75,76]. This study shows the direct bactericidal effect of the resulting composite (Figure 9). The bacteriostatic effect of nanocomposites with ZnO NPs may depend on the type of polymer matrix; however, differences in MIC values rarely exceed an order of magnitude [74,75,76,77]. This phenomenon may indicate that the antibacterial properties of ZnO NPs change insignificantly with a change in the type of polymer matrix. Therefore, ZnO NPs can be considered a universal dopant with antibacterial properties. The most commonly used polymer matrices are chitosan [74,78,79], starch [80], and thermoplastic synthetic materials [76,77,81]. It is also possible to use organo-silicon polymers with controlled physicochemical properties and biodegradable polymers [57,58]. A wide range of polymers “compatible” with ZnO NPs expands the range of possible applications of nanocomposites based on ZnO NPs. It should be noted that the bacteriostatic activity of ZnO NPs in the polymer matrix, including the fluoroplast that we used, can be comparable to or exceed that of “pure” ZnO NPs [82]. There are at least two possible explanations for this phenomenon. First, as mentioned above, the geometry of NPs is one of the factors that enhance their antibacterial activity [52,69]. It is likely that the shape of NPs depends on the method of synthesis. In most studies, spherical ZnO NPs were obtained by chemical synthesis methods, and they had a spherical shape [78,79,82,83]. Laser ablation makes it possible to obtain rod-shaped ZnO NPs with an expected higher antimicrobial activity [57,58]. Secondly, for fluoroplast, the ability to prevent the adhesion of microorganisms to surfaces treated with it has been described [21,33]. Interference with adhesion can be considered an additional mechanism of antibacterial activity. The release of NP clusters from the polymer matrix can be an additional way to enhance the antibacterial activity of nanocomposite materials [81]. Using the MIM method, we found that ZnO NPs form aggregates in the thickness of the fluoroplast polymer matrix (Figure 6). We assume that after prolonged use of the coating based on fluoroplast/ZnO NPs, the release of aggregates of ZnO NPs is possible, which can extend the antimicrobial effect of the composite. Verification of this assumption is a task for further research.

The cytotoxic properties of the composite material were also evaluated. It was found that on the surface of fluoroplast with the highest content of NPs (0.1%), the fibroblasts adhered unevenly and more sparsely, without forming a monolayer (Figure 10d). At the same time, there was no statistically significant decrease in the number of cells growing on the surface of the composite material per unit area (Figure 10a). The percentage of non-viable cells in the 0.001 and 0.01% fluoroplast/NPs ZnO variants did not change significantly compared to the control. The composite material supplemented with 0.1% ZnO tended to exhibit cytotoxicity (Figure 10b).

In a number of studies, the impact of zinc oxide nanoparticles, both in pure form and in the form of composite materials, was also evaluated (Table 3). In general, the use of ZnO NPs in polymer matrices, as well as doping with nanoparticles of other metals (Ag) [85], contributed to a decrease in the cytotoxic effect, up to its complete absence [86,87]. The use of biodegradable polymers such as PLA, PLGA, and PCL also made it possible to reduce the effect on cell growth and development in vitro [57,86,88]. It is interesting to note that many studies have shown significant toxicity for cancer cells, while for normal cells, the degree of effect was low [89,90]. In general, the results of our cytotoxic studies are consistent with the reported results; the effect on cell growth and development is comparable to the use of other biocompatible materials [57]. We also analyzed the cell nuclear area parameter (Figure 10c). As it is known, a change in the morphology of cell nuclei may indicate a change in the proliferative activity of the cell and is an indicator of disturbances in cell ion homeostasis, as well as oxidative stress [91]. However, the concentrations of ROS found by us (Figure 7) didn’t exceed several tens of nM, which is lower than the values associated with oxidative stress in eukaryotes [92]; therefore, oxidative stress cannot be considered a major mechanism of cytotoxicity. The investigation of additional potential mechanisms of cytotoxicity is a task for future research. The search for optimal concentrations of added ZnO NPs and/or methods for modifying the polymer matrix that provides a high antibacterial effect, but does not have a cytotoxic effect, is also the task of further study. The resulting material can be used for the antibacterial treatment of cutting boards, including those made with polymer, in the meat processing industry. In addition, the developed composite can be used in the prolonged antibacterial protection of work surfaces at the enterprise and the manufacture of packaging material.

## 5. Conclusions

A new composite material based on fluoroplast and ZnO NPs has been developed. Composite material fluoroplast/ZnO NPs made it possible to reliably fill micro damages in polymer cutting boards. The evenness of the surface and the absence of defects at the micro level were shown by the AMF method. The composite material affected the production of ROS (hydrogen peroxide and hydroxyl radical), 8-oxoguanine, and LRPS. It also possessed excellent bacteriostatic properties against both Gram-positive and Gram-negative bacteria. The functionalization of the polymer with ZnO NPs at a concentration of 0.1% resulted in a bactericidal effect, the suppression of growth, and the destruction of the biofilm structure. The developed coating does not show acute cytotoxicity against the primary culture of mouse fibroblasts. The resulting material is promising for the food industry, and in particular, the meat processing industry, and can be used for the antibacterial coating of work surfaces.

## Figures and Tables

**Table 1:** Results of microbiological studies.

Group	Time, h	Viable Bacterial Cells, CFU/mL			
		*P. aeruginosa*	*S. typhimurium*	*L. monocytogenes*	*S. aureus*
Control	6	500 ± 25	800 ± 40	96 ± 5	83 ± 4
	18	(8.0 ± 0.4) × 10^6^	(3.0 ± 0.2) × 10^6^	(3.0 ± 0.1) × 10^5^	(7.0 ± 0.4) × 10^5^
Fluoroplast	6	500 ± 25	700 ± 35	79 ± 4	78 ± 4
	18	(3.0 ± 0.2) × 10^6^	(6.0 ± 0.3) × 10^6^	(4.0 ± 0.2) × 10^5^	(2.0 ± 0.1) × 10^5^
Fluoroplast + 0.001% ZnO-NPs	6	10 ± 1	14 ± 1	12 ± 1	5 ± 1
	18	(5.0 ± 0.2) × 10^3^	(9.0 ± 0.5) × 10^3^	300 ± 15	200 ± 10
Fluoroplast + 0.01% ZnO-NPs	6	N/A	N/A	N/A	N/A
	18	(2.0 ± 0.1) × 10^4^	(4.0 ± 0.3) × 10^4^	500 ± 25	200 ± 10
Fluoroplast + 0.1% ZnO-NPs	6	N/A	N/A	N/A	N/A
	18	<5	<5	<5	<5

**Table 2:** Antimicrobial Properties of Polymer Composites and ZnO NPs.

No.	Polymer	Shape	NP Size, nm	Microorganism	MIC/MBC	Ref.
1	Chitosan	Sphere	50–70	*S. aureus*, *E. coli*	30 μg/mL/―	[74]
2	Gelatin	Sphere	~20	*P. aeruginosa*, *E. faecalis*, *C. albicans*	25–50 μg/mL/―	[75]
3	Polystyrene/ polyvinylpyrrolidone	Sphere	~5	*L. monocytogenes*, *S. enteritidis*, *E. coli*	100–500 μg/mL/―	[76]
4	Sodium alginate/ polyvinylalcohol	Sphere	162–164	*S. aureus*, *E. coli*	50 μg/mL/―	[77]
5	Polyethyleneglycol/ starch	Rod	40–1200	*S. aureus*, *E. coli*	81 μg/mL/―	[80]
6	Polymethyl-methacrylate	Sphere	100	*E. coli*	The bacteriostatic effect only with the background of exposure to plasma for 1 min	[81]
7	Chitosan, Hesperidin associated conjugate	Sphere	~29	*S. aureus*, *E. coli*	>6 mg/mL/―	[78]
8	Chitosan	Sphere	21–47	*S. aureus*, *B. subtilis*, *E. coli*	10 μg/mL/―	[79]
9	3-glycidyloxypropyl-trimethoxysilane	Sphere	~5	*S. aureus*, *E. coli*	Not reported	[83]
10	no	Sphere	20–25	*S. aureus*, *S. typhimurium*, *A. flavus*, *A. fumigatus*	20–100 μg/mL/―	[82]
11	Poly-lactic-co-glycolic acid	Rod	40–70	*E. coli*	10 μg/mL/―	[84]
12	Borosiloxane	Sphere/Rod	~90	*E. coli*	10 μg/mL/―	[58]
13	Fluoroplast	Rod	30–80	*L. monocytogenes,* *S. aureus,* *P. aeruginosa,* *S. typhimurium*	10 μg/mL/1 mg/mL	Our data

**Table 3:** The cytotoxic properties of ZnO-NPs and composite materials based on ZnO-NPs for eukaryotic cells reported in the literature.

No.	Composition	Size; Shape of ZnO-NPs	Cells, Cell Lines	Concentration	Effect	Ref
1	Nanofibers PCL (polycaprolactone), containing docetaxel loaded with ZnO	irregular	A549	1–500 μg/mL	Cytotoxicity against cancer cells	[93]
2	PVA/ZnO—NPs	50–150; flower	HFF	32–2000 μg/mL	NP concentration above 500 µg/mL significantly increased cytotoxicity	[94]
3	ZnO-Fe3O4 Composite	14–44, nanospheres	MDA-MB-231, NIH/3T3	1.5–200 μg/mL	Significant cytotoxicity against MDA-MB-231 cancer cells only	[89]
4	PLGA/ZnO-NPs	40–70, nanorods	SHSY-5Y	0.001–0.1 wt%	Toxicity has been shown at high concentrations	[84]
5	ZnO-NPs, Ag-doped–ZnO NPs	15–40, nanospheres	glioblastoma of the brain (U87)	1–500 μg/mL	Doping with ZnO Ag reduced cytotoxicity	[85]
6	PLA&PCL-ZnO-NPs	46–73	Fibroblasts	2, 4, 6 wt%	No cytotoxicity	[86]
7	PLGA/ZnO-NPs	~200, nanospheres	HepG2	0.000001–0.01 wt%	Cytotoxicity only at high concentrations of NPs (0.01%)	[88]
8	ZnO-NPs	9.26 ± 0.11	Hut-78 lymphoma T cell line	0–0.9 mM	Cytotoxicity is 1.5 times higher in samples with higher zeta potential and catalytic activity	[95]
9	ZnO-NPs	32.11 ± 7.659, nanospheres	HCT-116, Caco-2 and HEK-293	5–300 μg/mL	Higher cytotoxicity against cancer cell lines (HCT-116, Caco-2)	[90]
10	PMMA/ZnO-NPs nanocomposites		HeLa	0–100 mg/L	ZnO-NPS showed no cytotoxicity at concentrations up to 20 mg/L	[96]
11	Chitosan/PVA/ ZnO-NPs composite	15–35, nanocubes, (ZnO-NPs nanospheres)	L929	0.5, 1, 2%	Cytotoxicity not detected	[87]
12	Fluoroplast/ZnO-NPs	55–57, nanorods	Mouse lung fibroblasts	0.1 wt%	Moderate cytotoxicity	Our data

## Data Availability

The raw data supporting the conclusions of this article will be made available by the authors, without undue reservation.
